# Coronary artery calcium findings in asymptomatic subjects with family history of premature coronary artery disease

**DOI:** 10.1186/1471-2261-12-53

**Published:** 2012-07-17

**Authors:** Catalin Taraboanta, Cameron J Hague, GB John Mancini, Bruce B Forster, Jiri Frohlich

**Affiliations:** 1 Department of Pathology and Laboratory medicine, Faculty of Medicine, University of British Columbia, Vancouver, BC, Canada; 2 Department of Radiology, Faculty of Medicine, University of British Columbia, Vancouver, BC, Canada; 3 Healthy Heart Prevention Clinic, Providence Heart and Lung Institute, St. Paul Hospital, Vancouver, BC, Canada; 4 Department of Medicine, Faculty of Medicine, University of British Columbia, Vancouver, BC, Canada

## Abstract

**Background:**

To evaluate the frequency of positive coronary arteries calcium (CAC) scores in a unique population of asymptomatic first degree relatives (FDRs) of patients with angiographically confirmed early onset of coronary artery disease (CAD) and to assess their association with carotid ultrasound findings and other cardiovascular risk factors.

**Method and results:**

We scanned, using 64-slice multi-detector computed tomography, 57 asymptomatic FDRs (47 ± 9 years old; 44% male, 56% female), out of the 111 FDRs previously phenotyped for cardiovascular (CV) risk factors. The controls were 616 individuals (57 ± 10 years old; 76% male, 24% female) with no family history of cardiovascular disease, chest pain or diabetes selected out of the 3500 subjects scanned between 2002 and 2007. FDRs had higher risk of abnormal CAC scores compared to controls; odds ratio (OR) for the 75^th^ percentile was 1.96 (95% CI 1.04 – 3.67, p < 0.05).

**Conclusion:**

The frequency of abnormal CAC scores is two-fold higher in asymptomatic FDRs than in controls. CAC scan provides additional information on CV risk assessment in asymptomatic FDRs, particularly for those in the intermediate risk category.

**Clinical trial registration:**

NCT00387595

## Background

Validated family history of premature coronary artery disease (CAD) in men <55 and women <65 years of age is an independent risk factor for cardiovascular disease (CVD) [1-5]. Although Framingham risk score [6] does not include family history, European SCORE risk tools [7] and current Canadian and U.S. guidelines for management of dyslipidemia [8,9] stipulate up to a two-fold increase in the risk of developing CVD in the presence of positive family history of CAD.

Calcium deposits in the coronary arteries are considered a marker of atherosclerotic burden [10]. CAC assessed by computed tomography (CT) is a strong predictor of cardiovascular (Relative Risk [RR] = 9.6) and coronary events (RR = 11.1), non-fatal myocardial infarction (RR = 9.2), and an independent predictor of all-cause mortality [11-13].

Coronary artery calcification (CAC) score is recognized as a highly sensitive method to measure subclinical atherosclerosis in asymptomatic patients [11,12,14-16]. CAC scans provide incremental independent prognostic value above the Framingham risk factors in young asymptomatic men [16]. The benefits of defining a CAC scoring threshold to assess risk in asymptomatic individuals with confirmed family history of early cardiovascular disease remains uncertain.

We compared, using a prospective case – control design, the frequency of positive CAC findings, assessed by multi-detector computed tomography (MDCT), in asymptomatic individuals with and without family history of early CAD. In addition we compared CAC findings with carotid ultrasound indexes of early atherosclerosis (combined measurements of diffuse carotid intima-media thickening and focal plaques), as well as other traditional and non-traditional risk factors assessed in the first degree relatives (FDRs) cohort [17].

## Methods

The study protocol was approved by the joined institutional review board of St. Paul Hospital and University of British Columbia; all participants gave their informed consent.

### Study population

First degree relatives (FDRs) of patients with early onset CAD (men <50 years, women <60 years) confirmed angiographically as part of their clinical care were identified as the study cohort. The risk factor profiles and carotid ultrasound findings of the FDRs had been documented previously [17]. In brief, classical (age, gender, smoking status, blood pressure, lipid profile, apolipoprotein and fasting glucose) as well as newly recognized risk factors (lipoprotein(a), high sensitivity C-reactive protein and total homocysteine) had been measured. Carotid B-mode ultrasound was carried out in 111 FDRs with carotid intima-media thickness, plaque size and number recorded. Previously validated carotid ultrasound indexes, combining intima-media thickness and plaque measurements, were used to measure burden of atherosclerosis in both the carotid arteries of asymptomatic subjects [18,19]. Total plaque area (TPA) is the product of length and thickness of each focal lesion(s); total area (TA) is the sum of plaque areas and the area of diffuse, intima-media thickness measured, in the far wall of the common carotid artery; average total thickness (ATT) is the total area divided by the total length of carotid wall measured. The intima-media thickness was an average of measurements taken over a 10-mm, plaque free, arterial wall segment of left and right common carotid artery, within 2-cm proximity from the bulb. Millimeters and square millimeters were used to express measurements of distance and areas respectively.

Subjects who had a chest X-ray or CT in the previous 12 months, pregnant women or women who might have been pregnant were excluded from the study. Of the initial 111 subjects, a total of 57 consenting FDRs (2 parents, 18 children and 37 siblings), 25 years of age and older, without clinical CAD, diabetes or chest pain, underwent CAC scoring by 64-slice MDCT scan within one year of the carotid ultrasound and within a year and a half of the first contact.

Framingham risk scores were calculated and subjects classified at low, intermediate or high risk of having CV events based on current guidelines [9].

### Control population

Control subjects were drawn from 3500 asymptomatic individuals who underwent CAC scoring between January 2002 and May 2007 in the same medical imaging center. Exclusion criteria included presence of CAD, prior assessment of CAC (no follow-up examinations were included in the control group) or age under 40. Subjects were either self-referred, referred to the clinic by a physician or were executives having the scan done as part of their health plan. All subjects were required to submit a pre-scan questionnaire. Subjects reporting a history of chest pain, or not indicating an absence of chest pain, were excluded to ensure an asymptomatic cohort. To match the criteria applied to the study subjects we excluded control subjects that reported diabetes as a current health problem.

A total of 616 subjects (57.2 ± 10 years old; 76% men, 24% women) served as the control group. This represents 20% of the total number of subjects scanned, who stated, on the pre-scan questionnaire no family history of cardiovascular disease. Smoking status was confirmed in 416 subjects. At the time of the scan 43 were smokers (defined as currently smoking or gave up in the last month); extrapolating the results, smokers represent 10% of the control group.

### 64-Slice multi detector computed tomography

All FDRs were scanned with a 64-slice MDCT (Aquilion 64, Toshiba America Medical Systems, Tustin, California) while subjects from the control group were scanned with either 64- or a 8-slice MDCT (Lightspeed Ultra, GE, Milwaukee, Wisconsin). The participants were told to abstain from caffeine the morning of the day of the scan. No pharmacological intervention or oxygen was administered prior to the scan to reduce the heart rate, though 75 beats per minute was effectively used as the cut-off limit. The scan extended from the ascending aorta 12 cm inferiorly towards the cardiac apex with a slice collimation of 64 x 0.5 mm (100 mA, 120KVp, average effective dose 0.9-1.1 mSv) for the Aquilion and 8 x 2.5 mm (230 mA, 120KVp, average effective dose 1 mSv) for the Lightspeed Ultra. Breath-hold image acquisition used prospective ECG gating (triggered at 50% of cardiac cycle) to minimize radiation dose [20].

CAC scoring was performed by two physicians, in consensus, on an offline computer station using the VScoreTM with AutoGateTM (Vitrea, Vital Image Software package; version 3.9). The radiologists were not blinded when reading FDRs scans. Under radiologist control each coronary artery (left main, left anterior descending, left circumflex, right and posterior descending artery) was selected; Agatston and Volume scores were calculated by the software package. In calculating the Agatston score [21] plaques with greater attenuation were weighted higher (pixels 0.26-0.35 mm2; >130 Hounsfield units), while volumetric score using isometric interpolation eliminates partial volume effect [22]. The total Agatston and total Volume scores reported herein are the sum of partial scores obtained for the five coronary artery readings.

### Statistical analysis

Descriptive statistics for the study cohorts were presented as mean and standard deviation for all variables except total homocysteine, lipoprotein(a), high sensitivity C-reactive protein, carotid ultrasound measurements and CAC scores for which median and interquartile range were used. To test for the difference between means, Mann–Whitney rank test, which assumes independence of the two groups, was used. Fisher’s test and/or Chi-square were used to test for differences in dichotomous variables. Two tail tests and 95% confidence intervals were used for all analyses. The strength of correlation between the CAC (total Agatston and total Volume) score, other known risk factors and carotid ultrasound indexes in the FDRs was assessed by univariate analysis and Spearman’s rank correlation test. To determine whether family history of premature CAD is predictive of higher CAC scores after adjusting for age and gender we used a multivariate linear regression analysis. To determine if family history of early CAD increases the likelihood of positive findings at CAC scan we employed Chi-square analysis. We defined positive CAC scan findings as values over 75^th^ percentile for the appropriate age and gender [23,24]. The same 75^th^ percentile, age and gender adjusted, cut-off values were used to define positive carotid ultrasound findings [17]. To assess the agreement between the identification of the disease based on CIMT and CAC scores we employed the kappa measure of agreement [25].

Odds ratios with 95% confidence intervals were calculated, using a log-linear general model, to determine the odds of positive CAC findings in FDRs compared to controls. A p-value < 0.05 was considered statistically significant. All statistical analyses were performed using SPSS 12.0 (SPSS Inc., Chicago, IL, USA) software package.

## Results

### Clinical and biochemical characteristics of the cohorts

The FDRs were predominantly of European (48 out of 57) with only 6 of South-Asian, 2 of Chinese and one of Aboriginal background. There was a significant difference in the gender distribution between the study group (56% women, 44% men) and the control group (24% women, 76% men). FDRs group (47.3 ± 8.9 years old) was 10 years younger than the control group (57.2 ± 10 years, p < 0.001). The slightly higher incidence of smoking in FDRs (14%) compared to controls (10%) was not statistically significant. There was a higher incidence of CAC scores above the 75th and 90th percentile, age and gender adjusted values, in FDRs compared to controls (26.3% vs. 15.3% and 12.3% vs. 6.7%, respectively). A CAC score of 0 was recorded in 64.9% of the FDRs and 50.1% of the controls. Table 1 lists the biochemical and anthropometrical characteristics of the FDRs cohort (not available for controls).

**Table 1:** Characteristics of the first degree relatives (FDRs) cohort *

**n**	**57**
Age	47.3 ± 8.9
BMI	27.5 ± 4.5
Waist circumference (cm)	90.3 ± 15.2
Systolic BP (mm Hg)	117.5 ± 17.4
Diastolic BP (mm Hg)	74.9 ± 14.0
Fasting glucose (mmol/L)	4.92 ± 0.8
Total cholesterol (mmol/L)	5.43 ± 1.1
Triglycerides (mmol/L)	1.33 ± 0.8
LDL-C (mmol/L)	3.40 ± 1.0
HDL-C (mmol/L)	1.42 ± 0.43
TC/HDL-C	4.15 ± 1.48
Lp(a) (mg/L)	153 (43–467)
Total homocysteine (mg/L)	9.6 (8.8 - 11.0)
hs-CRP (mg/L)	0.9 (0.7 - 3.25)
Apo-AI (g/L)	1.54 ± 0.31
Apo-B100 (g/L)	1.04 ± 0.29
Smoking (n,%)	8 (14%)
Framingham risk score (%)	1% (1% – 5%)
**64-MDCT scores**	
Total Agatston score	0 (0–41.5)
Total Volumetric score	0 (0–50.5)
**Carotid Ultrasound Indexes**	
Average IMT (mm)	0.67 (0.62 - 0.73)
Total plaque area (mm^2^)	5.59 (0–21.34)
Average total thickness (mm)	0.77 (0.67 - 1.00)
Total area (mm^2^)	19.0 (13.45 - 35.44)
Plaque No (n)	1 (0–3)

* data is presented as mean ± SD, except for: homocysteine, hs-CRP, Lp(a), FRS and the imaging indexes were median and inter-quartile range was used.

### Correlations strength and likelihood of findings

The relationship between the extent of coronary calcification and the assessed risk factors including carotid ultrasound findings are summarized in Table 2. All carotid ultrasound measurements had a higher correlation with CAC scores than with any other risk factor including the Framingham risk score. Homocysteine, lipoprotein-(a) and high sensitivity C-reactive protein correlations with CAC scores were not statistically significant. In FDRs, kappa analysis of agreement showed number of plaques (kappa = 0.324, p < 0.05) and average total thickness (kappa = 0.226, p < 0.01), but not intima-media thickness (kappa = 0.031, p = 0.8) to be in agreement with the Agatston score findings above the 75^th^ percentile for the subjects age and sex.

**Table 2:** Correlations of total Agatston and total volume scores with risk factors and carotid ultrasound findings in FDRs

	**Total Agatston**		**Total Volume**	
	rho*	**p**	rho*	**p**
**n = 57**				
Age	0.425	0.001	0.421	0.001
Diastolic BP	0.267	0.045	0.267	0.045
Framingham risk score	0.279	0.036	0.280	0.035
fasting Glucose	0.298	0.017	0.283	0.024
Total Cholesterol/HDL-C	0.256	0.041	0.259	0.039
**Ultrasound indexes**				
AvgIMT	0.398	0.002	0.402	0.002
TPArea	0.458	0.000	0.460	0.000
AvgTThick	0.520	0.000	0.525	0.000
TArea	0.495	0.000	0.498	0.000
Plaque No	0.480	0.000	0.478	0.000

* rho - correlation coefficient (strength of correlation ranges form 0 to 1; 0 = no correlation); statistically significant p < 0.05; highly significant p < 0.005 (2-tailed). Note: Spearman's test measures the strength of correlations between calcium scores (in the heading) and other risk factors (listed in the first column) in the FDRs cohort; Carotid Ultrasound Indexes: AvgIMT = average intima-media thickness; TPArea = total plaque area; AvgTThick = average total thickness; TArea = total area; PlaqueNo = number of plaques.

The multivariate linear regression model, after adjustment for age and sex, found that family history of CAD was highly predictive of coronary calcium findings: total Agaston score (β = 0.102; p < 0.05) and total Volume score (β = 0.103; p < 0.05). To account for the CAC of 0 when assessing for the difference between cohorts both Agatston and Volume scores have been transformed in dichotomous variables based on the age and sex adjusted 75^th^ percentile cut-off point. If the 75^th^ percentile is used, FDRs are more likely (*χ*^2^ (1, 673) = 12.78; p < 0.000) to have positive CAC findings compared with controls (Table 3). Using the log-linear model we show that FDRs have higher odds of having positive CAC findings than controls; odds ratio (OR) is 1.959 (95%CI 1.044 - 3.673, p < 0.05). In sub-gender analysis, men with family history of early CAD had higher odds OR 2.460 (95%CI 1.024 – 5.9, p < 0.05) than women OR 1.806 (95%CI, 0.691 – 4.723, p = 0.228) to have positive CAC findings compared to controls.

**Table 3:** The predictive value of positive family history of premature CAD for coronary artery calcium findings

		**Total Agatston**		**Total Volume**	
Family history	Beta	Sig.	Beta	Sig.
Total †	0.102*	0.009	0.103*	0.011
Women &	0.185*	0.022	0.185*	0.022
Men &	0.053	0.227	0.051	0.267

* significant p < 0.05;† Multivariate linear regression model, age and gender adjusted; & age adjusted only.

### CV risk assessment

**Figure 1:**
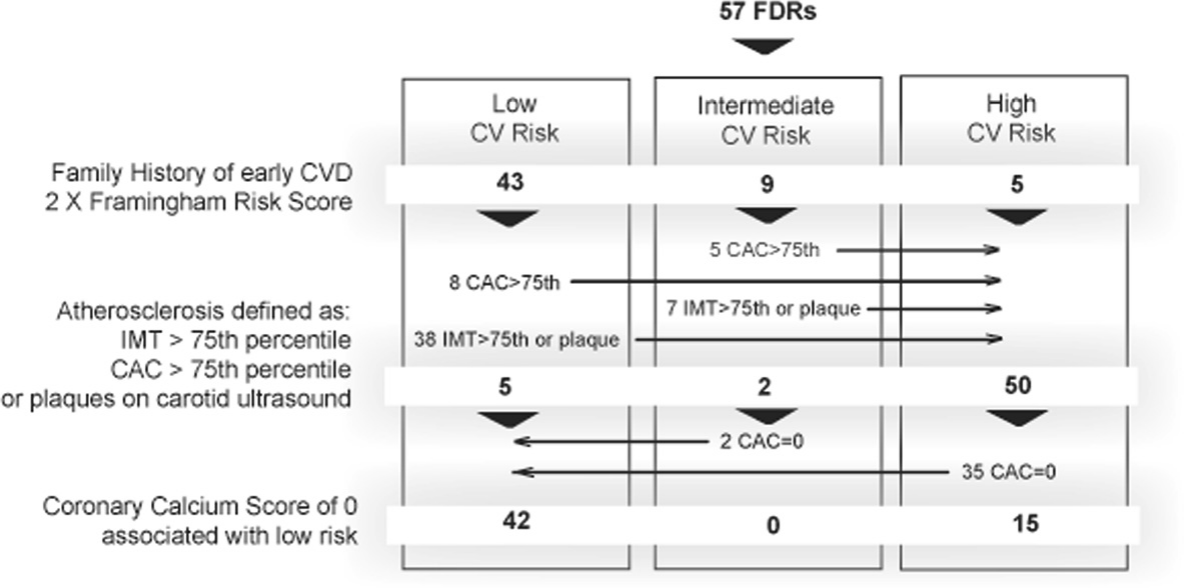
FDRs cardiovascular risk as defined by Framingham Risk Score, presence of subclinical atherosclerosis in either carotid or coronary arteries and negative calcium scoring scan*.

The 10 year risk of a CV event based on the Framingham risk score (FRS) [6] adjusted (two-fold) for the presence of family history of early CAD was low for 43, moderate in 9 and high for 5 FDRs [9]. Positive carotid ultrasound findings, IMT > 75^th^ percentile or presence of plaque, upgrades the CV risk category to high in 38 low risk and 7 intermediate risk FDRs, while CAC score >75^th^ percentile does the same for 8 low risk and 5 intermediate risk FDRs. CAC score of 0 downgrades 2 intermediate risk and 35 high risk FDRs to the low CV risk category (see Figure 1). In summary, subclinical atherosclerosis in either carotid or coronary arteries was confirmed in 45 out of the 57 FDRs, while a CAC score of 0 was found in 37 FDRs. The net result of using carotid ultrasound and CAC scoring, is reclassification of all subjects from the intermediate risk group according to Framingham risk factors into either high or low risk groups. 

## Discussion

Our study demonstrates that in asymptomatic subjects with a family history of early CAD the odds of having CAC above the 75^th^ percentile adjusted for age and gender is twice as high as in controls. While the result is similar to other studies it is important to point out that our study identified FDR’s not by questionnaire or subject recall but via the angiographic results of the proband, and also compares CAC findings to ultrasound indexes that assess both IMT and plaque burden [26]. The results indicate that these odds are higher in men than in women with family history of CAD when compared to controls. We also demonstrate significant correlations between CAC scan results and carotid ultrasound indexes of early atherosclerosis, particularly those that combine measures of focal plaque burden with measures of diffuse intima-media thickness measurements. Our study suggests that CAC scoring, in particular when considering CAC scores of 0, may further improve risk assessment in asymptomatic FDRs.

In the largest study to date that evaluated the impact of various risk factors on CAC findings, Budoff et al. showed that CAC can predict all cause mortality, independent of family history of premature CAD [13]. In the Framingham study cohort, a validated family history of CAD correlated with a two fold increase in CAC findings, when using the 90th percentile cut-of point in a younger cohort [27]. Independent of Framingham Risk Score (FRS), a CAC scan can improve assessment of risk of coronary events in subjects with FRS <10% but not for those with FRS > 10% [28]. In the Dallas Heart study, significant association between family history of myocardial infarction and CAC was shown in younger (men <45, women <55) but not in older subjects [29]. Similar to previous studies, our results indicate that in middle-aged (39–55 years) asymptomatic FDRs the incidence of positive CAC findings is double that of controls [26].

Terry et al. showed CAC (AUC 0.91) to be superior to IMT (AUC 0.73) as a predictor of angiographic CAD, findings suggested by Sesse et al. a decade earlier [30,31]. Recently, in the largest prospective study to compare CAC and IMT ability to predict CVD events in asymptomatic subjects, Folsom demonstrated that CAC is a better predictor of CHD while maximal IMT is a modestly better predictor of stroke [32]. In our unique cohort, the presence of family history was associated with a high odds ratio for detection of subclinical atherosclerosis using either CAC scan or IMT [17]. When the 75th percentile cut off is used, the CAC odds ratios are higher for men than for women. Furthermore, we have found that all subjects with family history of early CAD, where atherosclerosis had been identified by a positive CAC scan, also had an increased IMT or plaques by carotid ultrasound.

Taylor et al., in a prospective 4-year follow up study, demonstrated a significant and independent relation between carotid non-calcified atherosclerosis and progression of CAC. [16,33]. Our study demonstrated strong correlations between carotid ultrasound indexes and CAC score. Both CAC scoring and carotid ultrasound scans are used to evaluate burden of subclinical atherosclerosis, therefore a degree of overlap in their findings is to be expected. We’ve shown, however, that the overlap or agreement between the two methods for the detection of the presence of atherosclerosis in individual patients is modest, with a kappa coefficient of agreement between 0.2 and 0.3 on a 0 to 1 scale.

In practice, an Agatston score above 100 is considered to be highly predictive for CAD [34]. In our study, all 10 FDRs with an Agatston score over 100 had plaques detected at carotid ultrasound scan. However, not all subjects with carotid plaques alone or plaques and increased IMT, had positive CAC findings. Moreover, 22 FDRs with detectable carotid plaques, 13 of which had at least one or more cardiovascular risk factors (high blood pressure, dyslipidemia or smoking), had CAC score of 0. The fact that more FDRs had a CAC of zero compared with controls (64.9% vs. 50.1%) can be explained by the 10 years difference in average age between the cohorts. Thus, absolute scores like 0 and 100 which are not adjustable for age and sex were less useful for defining positive or negative findings than the 75^th^ percentile we’ve used.

While our study provides a unique look at asymptomatic FDRs of patients with early onset of angiographically documented CAD, and also compares CAC findings to ultrasound indexes that assess both IMT and plaque burden, there are several study limitations.

A number of differences between the study cohort and control population both in regards to data collected and demographics are present. As the control group was obtained retrospectively from a large set of patients having clinically indicated CAC scans, no CIMT data or blood work was obtained from the controls. As such, comment with regards to the nature of the CIMT data, outside of the correlation on a per patient basis in the FDR cohort, is limited. The same would hold true for the laboratory data (CRP etc.)The cohort is of predominantly of European ethnic background and thus the results may not be applicable to non-Caucasian populations. Additionally, there are significant age and sex differences between the FDR and control cohorts. While we have no information on other cardiovascular risk factors in the controls, the fact that they were refered for the scan suggests a clinical suspicion for CHD, most likely due to the presence of a major cardiovascular risk factor. This may explain the higher frequency of 0 CAC scores in FDRs and could have diminished the difference we noted in positive CAC findings between cohorts. It is well established that age and sex play a significant role in development of coronary artery calcium. While it would be inappropriate, based on the cohort differences, to directly compare raw Agatston or volume scores, it is felt that adjustment for age and sex, utilizing a percentile cutoff (75% in our study), as was done with the MESA data, helps to avoid this problem. Furthermore, a mixture of 8-and 64-slice MDCT scans were used in the control group. While an accepted source of error, large multicenter trials have demonstrated only negligible (less than 4%) differences between these two methods for assessing CAC [35]. As mentioned in the methods section, a lag time of up to one year between CIMT acquisition and CAC assessment may be present in select patients. Although in this setting we cannot entirely exclude progression of CAC values in this timeframe data from Min JK et al. determined progression of CAC to be a fairly slow process [36].

### Future directions

The initial presentation of up to 25% of patients presenting with CAD will be sudden death or unstable angina. Identifying the “at risk patient” has lead to an increased interest in adding imaging correlates to help guide risk stratification and ultimately risk factor modification.

Carotid ultrasound measures of plaque, CIMT and CAC have emerged as powerful tools to help accomplish this goal. Further data elucidating the relationship of CIMT and CAC in patients with a family history of early onset coronary disease are necessary.

Multiple studies have demonstrated a 10 year CV event rate close to 1% for subjects with zero Agatston score, thus such subjects should belong to the low risk category [12,37,38]. However, there is evidence that up to 10% of subjects with a zero CAC score can have clinically significant, minimally or non-calcified coronary plaques [39]. While in FDRs carotid ultrasound can identify all subjects with subclinical atherosclerosis, a CAC score of 0 may identify many FDRs who, despite some evidence of atherosclerosis based on carotid scanning, may actually have a low cardiovascular risk (see Figure 1), though this has not specifically been shown to be the case in any prospective studies. Moreover, the temporal evolution of carotid abnormalities and positive CAC findings may well be different, with the later requiring longer duration of disease.

In this patient population we have demonstrated that many patients with a CAC of 0 will have positive CIMT findings. However we have no control data to determine whether in a matched population this finding is constant. Whether those patients with a CAC of 0 but positive carotid ultrasound data represent a group that deserves more aggressive risk factor modification is not known.

Clinical trials investigating the validity of such approach in practice are warranted and given the differences in sensitivity for detection of disease between the two techniques noted in this unique cohort, such trials are especially warranted in FDRs.

## Conclusions

Asymptomatic FDRs have a two-fold increase in the likelihood of positive findings of CAC scoring compared to controls. CAC scores correlate highly with carotid ultrasound indexes of early atherosclerosis, but in individual patients, disparate results may be seen, Both methods provide additional information that is generally accepted to help clarify the risk of future CV events. Studies of the optimal use of these methods, especially in asymptomatic FDRs is warranted particularly in those perceived to be at intermediate risk based on currently used risk prediction algorithms that do not take into account findings from imaging studies.

## Competing interests

The authors declare that they have no competing interests.

## Authors’ contributions

CT participated in the collection and analysis of data and writing of the manuscript. CH participated in the data collection and writing of the manuscript. BF and JBM participated in conception of the study, supervision, data analysis and manuscript editing. JF participated in conception and oversight of the study, supervision, data analysis and manuscript preparation. All authors read and approved the final manuscript.

## Pre-publication history

The pre-publication history for this paper can be accessed here:

http://www.biomedcentral.com/1471-2261/12/53/prepub
